# Professional literacy that Taiwan’s bag manufacturing industry talents should possess: Analyzing from a practical perspective

**DOI:** 10.3389/fpsyg.2022.1032763

**Published:** 2023-01-13

**Authors:** Jing-Yun Fan

**Affiliations:** Department of Fashion Design, Tainan University of Technology, Tainan, Taiwan

**Keywords:** professional literacy, bag manufacturing industry, practical perspective, technical and vocational education, curriculum development, higher education institutions, on-the-job training

## Abstract

**Aim:**

This research aimed to explore the professional literacies that should be possessed by Taiwan’s bag manufacturing industry talents. This understanding will also help the technical and vocational education system to cultivate talents in Taiwan’s bag manufacturing industry.

**Methods:**

A qualitative research design was adopted. The outline of the interview on the professional literacy of Taiwan’s bag manufacturing industry talents was compiled, and seven interviewees were selected by purposive sampling from those who had worked (including supervisors) in Taiwan’s bag manufacturing industry for 21 to 42 years. One-on-one in-depth interviews with semi-structured interview questions were conducted. Using grounded theory, the content of the in-depth interviews was analyzed and discussed.

**Results and discussion:**

This study discusses what professional literacy of Taiwan’s bag manufacturing industry talents should possess from the practical perspective of Taiwan’s bag manufacturing industry leaders or senior managers. This study shows that bag manufacturing industry talents should possess knowledge, skills, and attitudes and values of common professional literacies. Moreover, bag designers, manufacturing technicians, patternmakers, hand stitchers, and production supervisors should acquire these professional literacies. The results of this study will help Taiwan’s bag manufacturing industry leaders or senior managers to highlight the importance of professional literacy that Taiwan’s bag manufacturing industry talents should possess.

**Conclusion:**

This study summarized the professional literacy of bag manufacturing industry talents into three major aspects: knowledge, skills, and attitudes and values. Based on this, suggestions are made as a reference for the implementation of curriculum development for talent cultivation in higher education institutions, and a reference for the learning and on-the-job training of bag manufacturing industry practitioners in the workplace.

## Introduction

The development of the bag manufacturing industry in Taiwan can be divided into three stages: the budding and developing period (1980), the growing and maturing period (1989), and the outward migration and transformation period (1991). Since the 1990s, due to the rise of the world factory in China, the world’s leading bag manufacturers have moved to set up factories in China to reduce personnel costs (Industrial Development Bureau, Ministry of Economic Affairs, Taiwan, 2014; Fan and Chien, 2019). Taiwan’s bag manufacturing industry is gradually transforming from traditional original equipment manufacturer (OEM) to brand creation in order to create higher economic output and profit. The main development direction is to cultivate the ability of original design manufacturers (ODM) and own branding and manufacturing (OBM) to create their own brands and establish brand image.

With the emergence of the knowledge economy, companies have started to place great emphasis on various rare resources and knowledge availability to enhance their competitive advantage and efficiency (Debrulle et al., 2013). Knowledge, skills, and competences are considered as a tool and intangible asset of a sustainable business or organization (Itami and Roehl, 2009). The intellectual capital of workers with knowledge, skills, and competences is becoming an increasingly important factor in creating corporate value and human capital is the most fundamental and important (Wuttaphan, 2017). Because employees’ knowledge, skills and competences are the source of innovation and insight (Hauschild et al., 2001), they are playing an increasingly important role in production and also have a great impact on the company. The employees’ learning and growth component is the most fundamental, as it affects the efficiency of the implementation of internal business processes (Díez-Vial and Montoro-Sánchez, 2014).

Wuttaphan (2017) also believes that competent and willing employees are the most important assets of a company. These studies point out the importance of employees’ knowledge, skills, and competences to a company. As members of the industry, Taiwan’s bag manufacturing industry practitioners have professional knowledge, skills, and competences, i.e., professional literacies, which provide enterprises with design and development, product production, and marketing skills/abilities. However, with the entry of developing nations into the labor market competition and the promotion of intellectual property rights protection, Taiwan’s bag manufacturing industry has lost its original competitiveness. In view of this, in the modern knowledge-based economy, what are the professional literacies required of the talents in Taiwan’s bag manufacturing industry? The time and effort of practitioners in different positions are limited, and it is not possible to place equal emphasis on all knowledge, skills, and competences or to put the same amount of effort into them. The importance of various types of knowledge, skills, and competences to the enterprise also varies. Therefore, it is necessary to identify the professional literacies that are more important to the enterprise and need to be strengthened.

To sum up, if the professional literacy of Taiwan’s bag manufacturing industry talents can be better understood, it would be helpful in three aspects: (1) Providing a reference for Taiwan’s enterprises to plan the content of training courses for bag manufacturing industry talents; (2) Helping Taiwan’s bag manufacturing industry talents understand which areas of expertise, skills, and competences they should enrich in order to make greater contributions to the industry; and (3) Helping the education sector grasp the knowledge, skills, and competences that should be cultivated in bag design and production education and then planning related courses for reference. These three aspects can also be considered as important contributions of this study. Therefore, the purpose of this study was to examine the following issues.

1. What are the common professional literacies that should be possessed by Taiwan’s bag manufacturing industry talents?
2. What are the professional literacies that should be possessed by the designers, manufacturing technicians, patternmakers, hand stitchers and production supervisors in Taiwan’s bag manufacturing industry?

## Literature review

### About literacy

Over the past few decades, scholars, adult literacy workers and program planners have proposed hundreds of definitions of “literacy” (Roberts, 1995), mostly focusing on technical skills in reading and writing related to school and academic progress (Mkandawire, 2018).

With the development of technology and information, literacy is described as the capability to recognize, explain, transmit and calculate applying visual, auditory, and digital materials that are relevant to different contexts (United Nations Educational Scientific and Cultural Organization, 2017). Lawton and Gordon (1996) defined functional literacy in an attempt to connect literacy with purpose. Some researchers later proposed critical literacy, utilitarian literacy and powerful literacy (Cambridge Assessment, 2013). Literacy involves a continuum of learning, a development of getting fundamental cognitive skills, using these skills that can help promote socio-economic development, constructing the capacity for social awareness and critical reflection to enable people to fully participate in their communities and wider society (United Nations Educational Scientific and Cultural Organization, 2005). At the psychological level, the act of literacy opens the door to meta-cognition and social consciousness, while literacy as a social cognitive act produces springboards for strategic perspective and pondering (Breed and Bailey, 2018).

The understanding of literacy continues to expand and develop, and literacy has changed into an intricate and vigorous notion that is constantly being explained, defined, developed, and debated in various ways (United Nations Educational Scientific and Cultural Organization, 2017; Zua, 2021). In addition to the primary connection to communication involving texts, stakeholders in other disciplines also use “literacy” to refer to the basic knowledge, skills and competences of the area of expertise, when literacy is related to other choice of words (such as functional literacy, information literacy, environmental literacy, and even professional literacy), the meaning of literacy becomes clearer because there is a context so it can be clarified (Mkandawire, 2018; Zua, 2021). In line with this, literacies are often used as shorthand for the capacity to access, understand, analyze, or evaluate these areas (United Nations Educational Scientific and Cultural Organization, 2017). Other researchers have suggested that literacy is skill, employment and competences in terms of merit and economy (Edwards and Potts, 2008). Literacy therefore means being able to use multiple skills to understand, build knowledge and communicate ideas.

In a broad sense, literacy refers to a body of knowledge, skills, and competence in a particular field or area (Mkandawire et al., 2019). Another broad definition of literacy is knowledge, competence and specialized skills in areas where people can demonstrate and apply unique abilities, perform tasks in a traditionally recognized and skilled manner and be accepted by a specified community as expressing truth and genuine information in that area. Some researchers define literacy as individual skills acquisition and empowerment, or describe literacy as social development, not as a static and impersonal state, but as a state that is individualized and enacted as a social practice (Benavot, 2015).

The workplace itself is becoming very different and automation, machine learning, and artificial intelligence (AI) will affect everyone in the coming years (Confederation of British Industry, 2019). Each individual must have the abilities of reading, writing, and numeracy, as well as the wider skills required by industry and employers, but professional knowledge, skills, and competences are unique to the individual and need to be acquired through training and experience (Mkandawire, 2018). Skills recognition, especially in terms of skills and job matching, has a key impact on the labor market (Braňka, 2016).

To summarize the above, this study examined the professional literacy of Taiwan’s bag manufacturing industry talents and therefore defined “literacy” as knowledge, skills, and competences focused on certain professional fields.

### Professional literacy main definitions

In this study, the researcher cited the concept of “professional literacy” as the professional knowledge, skills and competences required of Taiwan’s bag manufacturing industry talents. The following are the definitions of the relationship between the three categories proposed by the researchers and international organizations and the connotations of professional literacy that are relevant to this study.

Knowledge is an abstract concept that can be metaphorically packed, accumulated, and delivered like tangible objects from the concept of our mind works, which has been confirmed by cognitive scientists (Bolisani and Bratianu, 2018). For example, the iceberg metaphor has been widely used to explain explicit and tacit knowledge. Thus, knowledge flows like a fluid, from where it is created to where it is needed. Knowledge can be structured in the context of formal or informal learning (Rodrigues et al., 2021).

The World Economic Forum (2015) proposed to classify skills as foundational literacies, competencies, and character qualities, and stated that “Students require 16 skills for the 21st century”. Foundational literacies are the foundation on which more advanced and equally important competencies and character qualities are built, including literacy and numeracy, scientific literacy, information and communication technology [ICT] literacy, financial literacy, and cultural and civic literacy. Competencies are necessary to deal with complex challenges, including critical thinking/problem-solving, creativity, communication and collaboration. Character qualities are required to deal with changing environments, including curiosity, initiative, persistence/gift, adaptability, leadership, and social and cultural awareness.

The Organization for Economic Co-operation and Development (2018) proposed the key content of “the future of education and skills 2030,” with a view to developing transformative competencies that include the knowledge base, skills, and attitudes and values. A competence is defined as the ability to successfully meet complex demands in a particular context through the mobilization of knowledge (cognitive, metacognitive, socio-emotional and practical), skills, and attitudes and values (Taguma and Anger, 2016). Among them, knowledge is summarized as disciplinary knowledge, interdisciplinary knowledge, epistemic knowledge and procedural knowledge. Skills include cognitive and meta-cognitive skills (e.g. critical thinking, creative thinking, learning to learn and self-regulation), social and emotional skills (e.g. empathy, self-efficacy and collaboration), and physical skills (e.g. using new information and communication technology devices). Attitudes and values are divided into three levels of personal, local, social and global to face complex needs.

Skills and competences are frequently invoked by policy-makers in reference to labor market developments and education objectives; however, these concepts have different meanings in disciplines such as sociology, economics and education, and in the documents of the European Commission (EC) and the Organization for Economic Co-operation and Development (OECD). Therefore, the European Commission combined the task, skills and competences in 2021 and proposed a unified conceptual framework of tasks, skills and competences (Rodrigues et al., 2021).

Some researchers have proposed skills related to the psychological level, such as cognitive skills, non-cognitive skills, and character skills (Gutman and Schoon, 2013). “Non-cognitive skills” are relevant to a series of attitudes, behaviors, and policies such as motivation, perseverance, and self-control, thought to be fundamental to success in school and work. They are considered to be different from cognitive “hard skills” in areas such as literacy and numeracy, which are measured through academic examination. Non-cognitive skills are increasingly seen as equally, if not more influential, than cognitive skills in interpreting academic and employment achievement. Non-cognitive skills have an influence on certain outcomes in young people, but to date, there is limited causal evidence for an impact on long-term outcomes (Kautz et al., 2014).

Previously, World Economic Forum (2015) proposed character as a necessary skill for the 21st century, while Heckman and Kautz (2013) argued that character skills are able to compare to cognitive skills, with the view that character is not a trait but a skill, because character skill is stabilizing across duties at any age. They suggested that character is created by family, school, and social circumstances, and that skills can change throughout the life cycle. Skills development is a changeable procedure that sets the stage for later flourishing investments in the early years. Character skills include persistence (grit), believableness, concentration, self-control, self-respect and self-efficacy, toughness to hardship, empathy, humility, tolerance for different opinions, productive participation in society, etc. This set of character skills is similar to the non-cognitive skills proposed by Gutman and Schoon (2013) covering creativity, motivation, grit, self-control, self-awareness, social competence, toughness and coping, and meta-cognitive strategies.

The definition of professional literacy in this study is as follows:

1. Professional literacy is also defined as professional competence: This study aimed to investigate the bag manufacturing industry and the talent literacy required by employers, whose professional knowledge, skills, and competences are unique to the individual and need to be acquired through training and experience; therefore, professional literacy can be defined as professional competence from a utilitarian and economic point of view.
2. Professional literacy can be divided into knowledge, skills, and attitudes and values: The bag manufacturing industry talents discussed in this study, in the bag manufacturing area or profession, need the extensive scope of professional knowledge, skills, and attitudes and values.

### Occupations and professional literacy related to the bag manufacturing industry

#### Occupations related to the bag manufacturing industry

The bag manufacturing processes are broadly classified as designing, selecting materials, pattern-making, cutting and preparing components, assembling and checking, and packing (Industrial Development Bureau, Ministry of Economic Affairs, Taiwan, 2014). Accordingly, the researcher relied on the edited *Standard Occupational Classification of the Republic of China* (Rev.11) of Directorate General of Budget, Accounting and Statistics of Executive Yuan, Taiwan (2021), and the European Skills, Competences, and Occupations (ESCO) website of European Commission (2022), occupational definition and alternative labels as shown in Table 1.

**Table 1:** Bag manufacturing industry related occupations list.

**Occupations**	**Occupational definition**	**Alternative labels**
Product and fashion designer	Personnel engaged in product and fashion design and development for the manufacturing industry.	• Fashion designer • Accessory designer • Leather goods designer
Textile and leather craft workers	Craftsmen who use traditional handicraft techniques and patterns to make fabrics, and traditional handbags and other accessories.	• Fabric Craftsmen • Leather goods artisanal worker • Leather goodsmanufacturingtechnician • Leather goods hand stitcher
Apparel proofing and tailoring personnel	Worker who is engaged in proofing, drawing and cutting of products related to fabrics, leathers and other materials.	• Bag sample maker and cutting operator • Leather goodspatternmaker • Leather goods CADpatternmaker
Shoe manufacturing and related workers	Workers engaged in the manufacture and repair of various natural or synthetic leather products.	• Leather goodsmaintenance technician • Leather goodsmanufacturingtechnician • Leather goods qualitymanager

#### Professional literacy for bag manufacturing industry talent

According to the *Occupational Outlook Handbook: Fashion Designer* published by the Bureau of Labor Statistics, United States Department of Labor (2022), accessory designers create original accessories, and design and manufacture items including belts, hats, handbags, suitcases, scarves, etc. They must have professional literacy such as sketching designs, choosing fabrics and patterns, and instruction on how to make the products they design. In addition, they must be creative and apply critical thinking/problem-solving in their job responsibilities and be able to communicate and collaborate with others.

Another example is the ESCO website of the European Commission (2022), which showed that a leather goods designer must be responsible for the creation process of leather goods, analysis of fashion trends, following market research and forecasting demand, materials and producing drawings and sketches. The leather goods designer determines the range of materials and components, defines the design specifications and works with technical teams.

Taiwan’s bag manufacturing industry talents come from diverse academic backgrounds, including industrial design, product design, and fashion design. Industrial designers have functions that are highly similar to those of the bag manufacturing industry design professionals, which are the crystallization of art, technology and science (Findeli, 2001). Komnenic et al. (2016) also mentioned that industrial designers should have knowledge, skills and competencies and that industrial design requires expertise not only in design and engineering, but also in cross-domain integration.

From the above literature, it can be concluded that the professional literacy of bag manufacturing industry talents requires knowledge, skills, and attitudes and values. The knowledge includes material characteristics, product structure knowledge, etc. Skills include drawing skills, production skills, computer drawing skills, tool application skills, trend analysis, etc. Attitudes and values include creativity, communication skills, learning skills, career planning skills, professional attitude, etc.

## Research design

### Grounded theory

This study is grounded theory oriented for the reason that grounded theory provides descriptions and explanations that are designed to create notional prototypes that may be continuously transformed into additional hypotheses (Charmaz, 2014). Therefore, this approach should be suitable to learn about the development and present status of the bag manufacturing industry in Taiwan from the perspective of those in charge of the manufacturing industry or senior managers and the common professional literacies required of those working in the industry and the professional literacies required of those in different positions, including knowledge, skills, and attitudes and values.

### In-depth interview

This study used in-depth interviews as the data collection method because the interview method can provide an in-depth understanding of the actions, mentality and the reasons and meanings behind the researched person.

### Instrument

After summarizing and analyzing the literature, the researcher developed the first draft of the question guide for the semi-structured interview and then discussed it with experts and scholars specializing in professional literacy to enhance the validity of the experts. The question guide for the semi-structured interview is shown in Table 2. After the preliminary interviews were conducted to explore the suitability of the interview outline proposed in this study, it was then revised by experts and scholars in the industry and academia.

**Table 2:** Question guide for the semi-structured Interview.

**No.**	**Research topics**	**Questions asked**
1	Taiwan’s bag manufacturing industry talents’ common professional literacies	What common professional literacies should be possessed by Taiwan’s bag manufacturing industry talents?
2	Bag designers’ professional literacies	What professional literacies should be possessed by designers in Taiwan’s bag manufacturing industry?
3	Bag manufacturing technicians’ professional literacies	What professional literacies should be possessed by manufacturing technicians in Taiwan’s bag manufacturing industry?
4	Bag patternmakers’ professional literacies	What professional literacies should be possessed by patternmakers in Taiwan’s bag manufacturing industry?
5	Bag hand stitchers’ professional literacies	What professional literacies should be possessed by hand stitchers in Taiwan’s bag manufacturing industry?
6	Bag production supervisors’ professional literacies	What professional literacies should be possessed by production supervisors in Taiwan’s bag manufacturing industry?

### Determination of interviewing companies and interviewees

In this study, purposive sampling was adopted to select companies in the bag manufacturing industry and according to the research questions and objectives, research participants who could provide a significant amount of information on the research questions were selected as interviewees (Patton, 2014; Liamputtong, 2019).

The decision of the interviewees was based on theoretical sampling principles. At the beginning of the study, the researcher did not determine the list of companies to be interviewed or the number of companies to be interviewed. However, the first interviewed company (hereafter referred to as Company A) was selected based on the intensity sampling principle of selecting excellent or rich cases with high information density and intensity, rather than selecting very unusual cases for the study (Patton, 2014; Benoot et al., 2016). Established in 1977, Company A is a bag manufacturer with many years of experience and has been certified by the International Organization for Standardization (ISO), and the Business Social Compliance Initiative (BSCI) Code of Conduct to ensure quality management and social responsibility. The researcher had a semi-structured in-depth interview with the person in charge of Company A to compile and extract the interview data to provide a theoretical framework for the development of this study.

After the first interview, the researcher then searched for companies in the manufacturing industry related to the core concept to enrich the data. Eisenhardt (1989) pointed out that 4–10 cases are usually well employed to produce more complex theories. All seven interviewees were required to be in charge or to be senior managers in the bag manufacturing industry in Taiwan, in order to be representatives. The information of the interviewees is shown in Table 3.

**Table 3:** Individual background and experience of each interviewee.

**Interviewee (code)**	**Sex**	**Educational background**	**Position**	**Experience working in the bag industry (years)**
A	Male	College	Person in charge	42
B	Male	Master’s	Person in charge	21
C	Female	Master’s	Manager	27
D	Male	High school	Person in charge	34
E	Male	Doctorate	Person in charge	36
F	Male	Bachelor’s	Person in charge	36
G	Male	Master’s	Person in charge	21

### Collection and analysis of data

The data for this study were collected from semi-structured in-depth interviews with seven interviewees. The researcher conducted one-on-one, face-to-face interviews and recorded the interviews for later transcription, with each interview lasting approximately 1–2 h.

In grounded theory, data collection and analysis are interrelated processes, starting with the first subset of data which guides the collection of the next phase of data (Corbin and Strauss, 2014). The purpose of the text analysis of the grounded theory is to acquire the professionalism of Taiwanese bag industry talents. In this stage, the text analysis, open coding, axial coding, and selective coding were carried out sequentially.

### Coding

The researcher invited two independent coders to assist with coding, to help the coders familiarize themselves with the topics, and understand the coding categories and ensure that all coders understood the meaning of the categories. Then, a small number of samples were selected as samples for testing the coding. During the testing coding process of some samples, each coder needed to code independently and could not discuss or guide each other.

After completion, the researcher invited qualitative research experts, interviewee B and interviewee E to participate in triangulation to confirm that the two coders had achieved the desired reliability, and then formal coding could be started. First, all the interviewed verbatim texts were coded into free categories and gradually open codes were formed. Then each open codes of the same concept were grouped into higher-level axial codes domain context concept. These contexts, intervening conditions and outcomes all relate to one primary domain, and the interconnections form a set of paradigmatic models of value concepts (Glaser and Strauss, 2006), culminating in selective codes.

In this study, the researcher systematically collected and analyzed data from the above process to organize open codes, axial codes, and selective codes for each research topic shown in Table 2. In addition, to protect the privacy of the interviewees, the interview data are coded in the text, where the first code is the interviewee code and the seven interviewees are indicated by “A, B, C, D, E, F, and G”; the second code is the question number and the third code is the concept. Take “A-2-1” as an example; it means “A interviewee - second question - first concept.”

### Reliability and validity

#### Reliability

In order to achieve dependability (also internal reliability) in this study, triangulation was used in data acquisition, coding, investigator, and theory to confirm that similar results were obtained. The researcher conducted the results and discussions of this research based on open codes, axial codes and selective codes and carried out an external audit trail after completion (Gray, 2014). The researcher invited qualitative research experts, interviewee B and interviewee E to check whether the research findings identified by the researcher were verifiable, logically derived, and grounded in the collected data. The procedure and results are presented in their entirety to allow the reader to determine whether the study can be replicated within the limits of the natural context.

#### Validity

In order to achieve transferability (also external validity) in this study, triangulation was used in bringing together different sources of data to converge or conform to one interpretation and comparing data from different sources in a rigorous manner to confirm the conclusions.

Credibility (also internal validity) is achieved through the use of transferring the researcher’s expert status with the interviewee (Goulding, 2011) and carrying out the “member checking” procedure. Interview scripts or summaries of the researcher’s findings were sent to the interviewees to express their comments, in order to find out whether the interviewees agreed with everything they had said in the interview. Finally, an independent qualitative research expert checked all the data collected in the research process to establish an external audit trail for relevant issues (de Kleijn and van Leeuwen, 2018).

## Results and discussion

### Taiwan’s bag manufacturing industry talents’ common professional literacies

**Figure 1:**
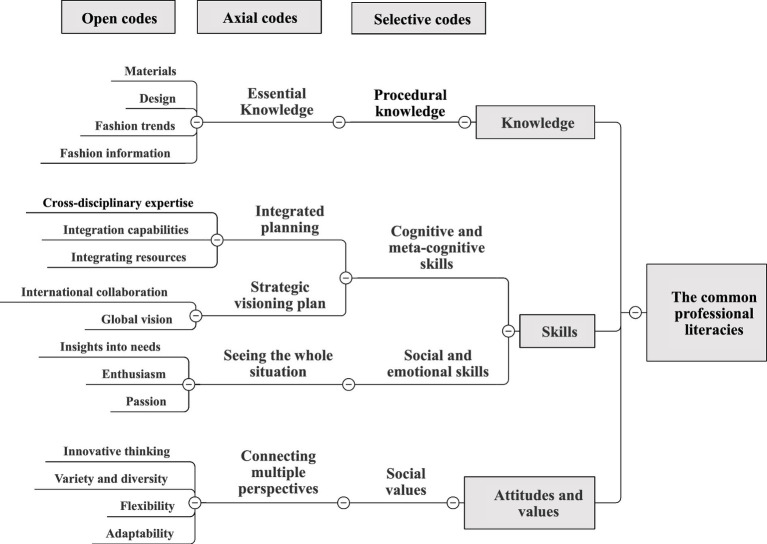
Taiwan’s bag manufacturing industry talents’ common professional literacies coding.

The following analysis of the common professional literacies that should be possessed by Taiwan’s bag manufacturing industry talents are shown in Figure 1.

In terms of knowledge, Taiwan’s bag manufacturing industry talents should have procedural knowledge about the necessary knowledge of bags (e.g., materials, design, fashion trends, and fashion information). Interview comments include:

*The design ability of a company will attract customers to place orders*. (A-1-1)

*The most important thing is design*. (E-1-1)

In terms of skills, Taiwan’s bag manufacturing industry talents should have cognitive and meta-cognitive skills (e.g., cross-disciplinary expertise, integration skills, resource integration, international cooperation, and global vision) in order to integrate different professional fields, resources, and international perspectives, understand international affairs, and plan the company’s strategic visioning plan to cooperate with companies around the world. Talents should also have social and emotional skills (e.g., insight into needs, enthusiasm, and passion), be passionate about their work and be enthusiastic and sincere in understanding consumer needs. Interview comments include:

*You should understand the key skills of sewing, pattern making and design, as well as the use of materials and fashion changes in the market*. (A-1-2)

*Materials and popular information are very important*. (C-1-4)

*Integrate diverse information and cross-disciplinary expertise*. (D-1-7)

*The common competency is insight into consumer and market needs*. (G-1-8)

*Integrate resources and to cultivate the ability to work together internationally*. (G-1-9)

In terms of attitudes and values, Taiwan’s bag manufacturing industry talents should have a passion for their work and social values (e.g., innovative thinking, diversity and variety, and flexibility and adaptability) that link innovation, diversity, and variety with a cross-disciplinary perspective. Interview comments include:

*The main thing is to have enthusiasm for the job*. (G-1-8)

*Innovative and flexible thinking is the only way to lead a brand*. (G-1-3)

In summary, the knowledge required for Taiwan’s bag manufacturing industry talents is the procedural knowledge of essential knowledge of manufacturing industry-related duties, which enables the development of a structure for design thinking and systems thinking to identify and solve problems (Organization for Economic Co-operation and Development, 2019a). In terms of skills, the cognitive and meta-cognitive skills of integrated planning cover cross-disciplinary expertise, integration skills and integration of resources, a phenomenon in line with Manzini’s (2014) view. Manzini (2014) stated that designers learn to collaborate with others within the framework of social resources, project participants and stakeholders are integrated and enhanced when they are responsible for highly dynamic action projects. In addition, if Taiwan’s bag manufacturing industry can cooperate internationally, it shows that this manufacturing industry is satisfactory and can be considered an influence with great communication condition, connection and operate group teamwork approval and achievement (Lowry et al., 2009). Taiwan’s bag manufacturing industry talents with insightful, enthusiastic, and passionate social and emotional skills will respect others and put themselves in the shoes of others (Berger and Frey, 2015). Innovative thinking, change and diversification, flexibility, adaptability in connecting multiple perspectives of attitudes and values is all necessary to adjust to high-speed technological progress. Workers must obtain social skills, as well as knowledge and attitudes and values. In order to remain effective, workers need to obtain the newest knowledge and skills throughout their working careers, which should be open, have a good attitude toward long-established learning and curiosity (Berger and Frey, 2015; Organization for Economic Co-operation and Development, 2019b,c).

### Bag designers’ professional literacies

**Figure 2:**
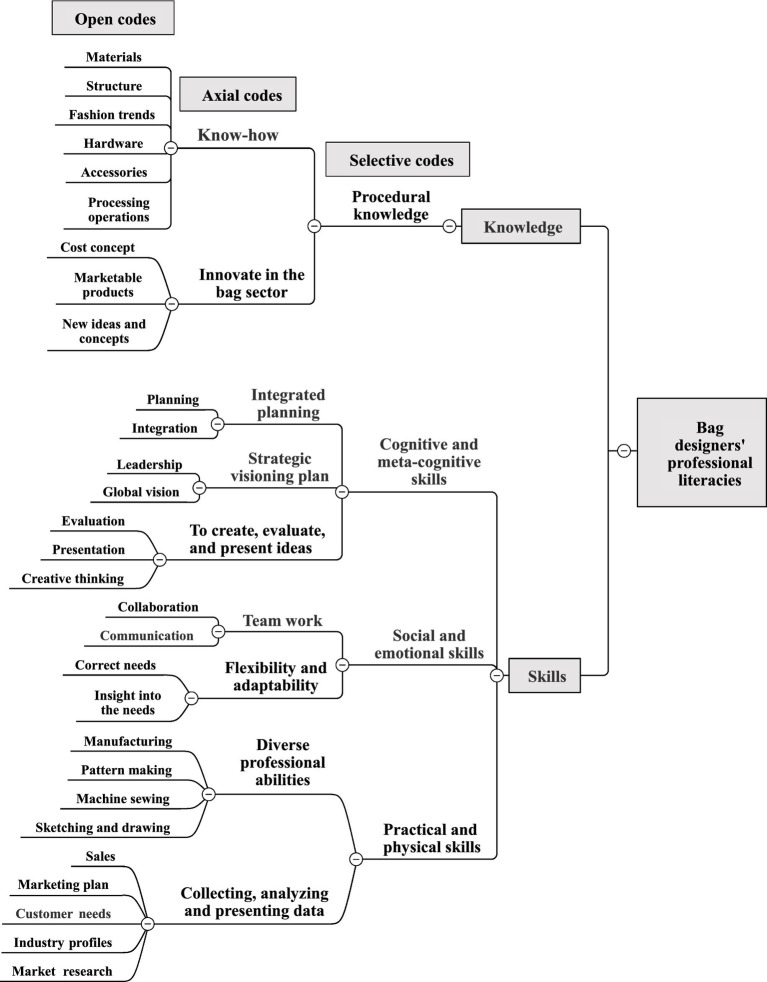
Bag designers’ professional literacies coding.

The following is an analysis of professional literacies that should be possessed by designers in Taiwan’s bag manufacturing industry as shown in Figure 2.

In terms of knowledge, the main focus is on the procedural knowledge of bags (e.g., bag materials, structure, hardware, accessories, processing operations, fashion trends, cost concept, new ideas and concepts, and marketable products). The bag designers need to understand this knowledge in order to be able to direct their creativity to the bag field and to be able to control the cost and design products that meet the market demand. Interview comments include:

*We go to department stores to find brand-name handbags or go to the market to find styles and then modify them to meet the needs of our customers*. (E-2-7)

*Have a deep understanding of the proper terms of bags, materials, structure, hardware, accessories, processing operations, fashion trends, etc*. (G-2-8)

*To know the cost and at what price you want the goods to be sold*. (G-2-10)

Skills can be divided into cognitive and meta-cognitive skills (e.g., integration, planning, leadership, global vision, creative thinking, evaluation, and presentation), social and emotional skills (e.g., insight into requirements, modification of requirements, effective communication, and collaboration), practical and physical skills (e.g., sketching and drawing techniques, machine sewing techniques, manufacturing techniques, pattern making techniques, market research, customer demand, and industry profile). Accordingly, bag designers with an international outlook and creativity are able to integrate different thematic concepts, propose a strategic visioning plan to meet the market demand and then create, evaluate, and present various effective ideas. Interview comments include:

*We need to understand bag manufacturing, pattern-making and sewing. In addition, we should understand fashion trends, market changes and application materials*. (A-2-1)

*Planning skills, teamwork, communication and working with people with different traits are also important*. (A-2-2)

*In addition to personal creativity, they should also have the core competencies of manufacturing technicians and patternmakers, and they should know some machines and equipment*. (B-2-3)

*Designers must have graphic design skills*. (C-2-4)

*Designers must understand sketching and drawing and have an international perspective and creativity, in order to meet the market demand and plan the strategic visioning plan*. (C-2-5)

*I think we need to get more diversified information*. (D-2-6)

*Go to the market to find something and understand customer needs*. (E-2-7)

*We understand the relationship between cost and the bag production process, materials and techniques, and must always meet the market demand*. (G-2-10)

*In addition to insightfulness, you should understand the design and pattern-making process, bag structure, finished product sales and other sales channels*. (G-2-11)

In summary, the knowledge required by bag designers is mainly related to the procedural knowledge of bags, which enables the development of a structure for design thinking and systems thinking to distinguish results and overcome problems (Organization for Economic Co-operation and Development, 2019a). In terms of skills, the cognitive and meta-cognitive skills required are constructive innovation, the bag designers’ ability to incorporate new, novel inspirations and images and apply them to present or desirable situations (Taguma and Anger, 2016; Bialik and Fadel, 2018). Social and emotional skills of communication and collaboration allow bag designers to build good interpersonal relationships at work (Heckman and Kautz, 2012; Organization for Economic Co-operation and Development, 2019b) and complete the tasks assigned by the company; one of the practical and physical skills is manual skills, which must adopt and manage objects, a set of tools or devices and artefacts to achieve specific results (Organization for Economic Co-operation and Development, 2019b); also, affiliated with collecting, analyzing and presenting skills capability of market research, customer demand, business, industry profile, and marketing plan, they need to conduct market research, interviews, or big data analysis for digital literacy, including collaboration and communication, information and data literacy, digital formation innovation, security, and problem solving (Vuorikari et al., 2016).

### Bag manufacturing technicians’ professional literacies

**Figure 3:**
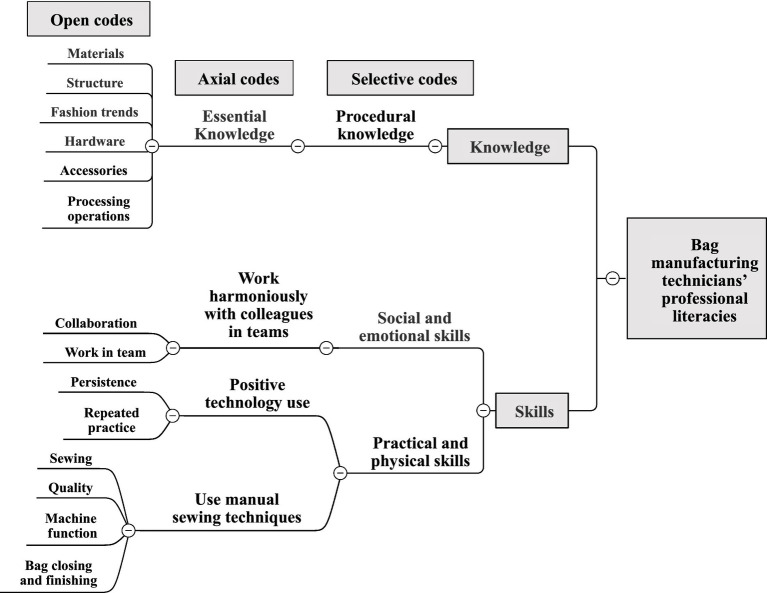
Bag manufacturing technicians’ professional literacies coding.

The following analysis of professional literacies that should be possessed by manufacturing technicians in Taiwan’s bag manufacturing industry can be seen in Figure 3.

In terms of knowledge, manufacturing technicians should have procedural knowledge about bags (e.g., bag materials, structure, hardware, accessories, processing operations, and fashion trends). Interview comments include:

*They should require familiarity with material characteristics, such as types of leather and fabrics and knowledge of bag trends.* (A-3-1)

*They must understand bag processing operations and countertop work, gluing, hardware installation, etc.* (B-3-3)

*Have a deep understanding of the proper terms of bags, materials, structure, hardware, accessories, processing operations, fashion trends, etc*. (G-3-8)

In terms of skills, manufacturing technicians should possess both social and emotional skills (such as collaboration for working in teams) and practical and physical skills (such as sewing, machine functions, bag closing and finishing, quality, persistence, and repeated practice). Only in this way can they work harmoniously with colleagues in teams in the bag manufacturing industry and then use hand-operated sewing and stitching approaches to produce or repair fabrics or textile-based objects. They should persist and practice continuously to improve the production techniques. Interview comments include:

*We have skills in processing various bag-making machines (including some computerized sewing machines)*. (B-3-2)

*Adjust and calibrate various bag-making machines*. (B-3-6)

*You should keep practicing to improve your production skills*. (D-3-3)

*The most important thing is sewing techniques*. (E-3-4)

*Bag closing and finishing techniques are the most important and difficult skills for manufacturing technicians*. (E-3-5)

In summary, the knowledge required by manufacturing technicians is the knowledge of the bag of procedural knowledge, for example, processing operations is the knowledge of “how” (Organization for Economic Co-operation and Development, 2019a), which helps to solve complex problems in manual or machine technology and allows the transfer of other knowledge and the connection of other attitudes and abilities (Benander, 2018). In terms of skills, social and emotional skills of collaboration and working in teams, by understanding demands, opinions of others as well as be able to handle conflicts in the team and facilitate collaborative and participatory problem solving (Taguma and Anger, 2016). Practical and physical skills are mostly manual skills, which must adopt and manage objects, a set of tools or devices and artefacts to achieve specific results (Organization for Economic Co-operation and Development, 2019b). There is also persistence and repeated practice skills that help manufacturing technicians improve their technical expertise and enable them to understand that it is necessary to make an effort to persist after failure and be able to succeed (Pourdehnad et al., 2011).

### Bag patternmakers’ professional literacies

**Figure 4:**
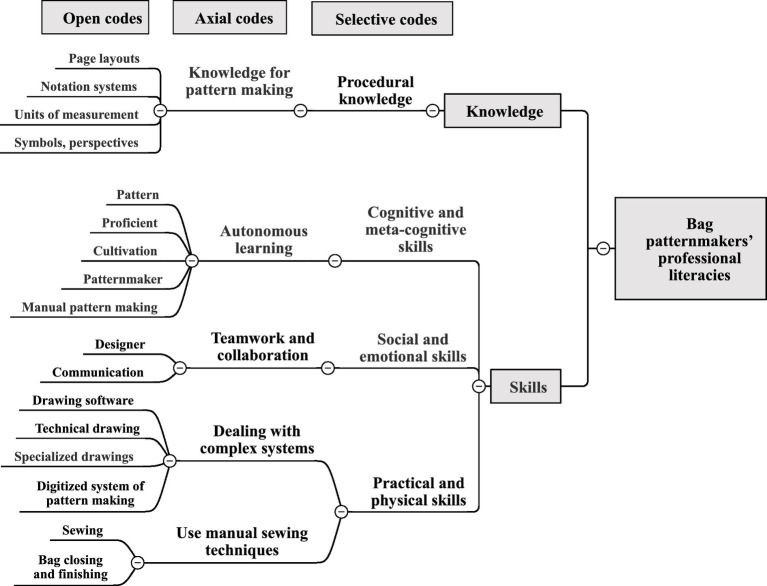
Bag patternmakers’ professional literacies coding.

The following is an analysis of professional literacies that should be possessed by patternmakers in Taiwan’s bag manufacturing industry (shown in Figure 4).

In terms of knowledge, patternmakers should have procedural knowledge of bag pattern making (such as symbols, perspectives, units of measurement, notation systems, page layouts, and fashion trends) to help make technical drawings of bag patterns. Interview comments include:

*They should require familiarity with bag pattern making expertise such as symbols, perspectives, page layouts, notation systems, and units of measurement*. (B-4-1)

*You should keep promoting your bag pattern making symbols, perspectives, and page layout expertise.* (D-4-3)

*Familiar with the symbols, page layouts, and units of measurement in digitized systems of pattern making.* (E-4-4)

*Understanding of digitized systems of pattern making, and familiarity with complex systems such as symbols, notation systems, and units of measurement are necessary.* (G-4-3)

In terms of skills, patternmakers should possess both cognitive and meta-cognitive skills of autonomous learning (e.g., self-training, skilled manual pattern making). They need to develop social and emotional skills of teamwork and collaboration (e.g., collaboration, and communication with designers) so as to understand the manufacturability and properties of the bag and complete the patterns required by the design. In addition, to be proficient in manual pattern making, patternmakers should also have the sewing ability of bag closing and finishing, as well as the practical and physical skills of handling complex systems (such as technical drawing, specialized drawings, drawing software, digitized systems of pattern making, sewing, and bag closing and finishing). Interview comments include:

*Because sewing is related to the bag patterns it can be turned up to become a complete bag*. (A-4-2)

*Communicate fully with designers and work together as a team to maintain a good understanding*. (B-4-2)

*They must have the skills of sewing and bag closing and finishing, completing patterns, and making finished bags by themselves*. (B-4-2)

*Have the ability of both manual pattern making and computer pattern making*. (E-4-1)

*Start from cutting patterns, and then from the simple bags they design the pattern, step by step. Besides, natural talent, and continuous independent learning, understanding and familiarity with manual pattern making are also very important*. (E-4-2)

*To be proficient in manual pattern making to use digitized systems of pattern making, and familiarity with complex systems*. (G-4-2)

In summary, the knowledge required by patternmakers is to understand the manual pattern making system of pattern-making and the procedural knowledge of how digitized systems of pattern making work, which helps patternmakers differentiate and comprehend the ill-defined equipment of the actual world (Benander, 2018). In terms of skills, self-training of cognitive and meta-cognitive skills, skilled manual pattern making, self-regulation and learning-to-learn skills, are also widely regarded as key competencies for long-established learning (Kikas and Jõgi, 2016). Social and emotional skills of collaboration and communication enable patternmakers to better define personal targets, handle personal emotions, strengthen interpersonal connections, and gain productivity (Payton et al., 2008). With influential communication, all participants can reach their targets, at least partially or during the interaction (Taguma and Anger, 2016). Practical and physical skills include using the newest ICT devices in addition to manual skills, and manual pattern making is related to manual dexterity and craftwork (Organization for Economic Co-operation and Development, 2019b). Patternmakers also need to have the skills to use computerized patterning systems, which are part of product development, to convert design output into complete manufacturing facilities to produce a sufficient number of products (Taguma and Anger, 2016).

### Bag hand stitchers’ professional literacies

**Figure 5:**
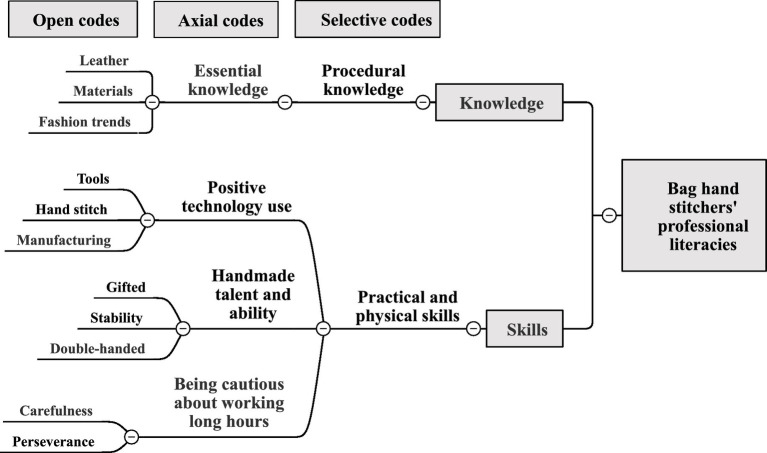
Bag hand stitchers’ professional literacies coding.

The following is an analysis of professional literacies that should be possessed by hand stitchers in Taiwan’s bag manufacturing industry, as shown in Figure 5.

In terms of knowledge, hand stitchers should have professional procedural knowledge of leather, materials, and fashion trends to be able to make good use of the characteristics of materials when hand stitching fashion bags. Interview comments include:

*They should require familiarity with material characteristics, such as types of leather and fabrics, and knowledge of bag trends*. (A-5-1)

*Such things as table works, gluing, hardware installation, etc. are all part of the bag’s manual process*. (B-5-3)

In terms of skills, hand stitchers should have practical and physical skills (such as natural talent, stable hands, tools, manufacturing, hand stitching, care, and perseverance). Hand stitchers must have dexterous hands to work long hours with patience and care in order to hand-stitch perfect, delicate bags with tools such as needles and thread. Interview comments include:

*The ability to hand stitch is an instinct that comes from a steady hand, combined with acquired effort*. (A-5-1)

*Hand stitchers are more delicate and require patience and care to make bags by hand for a long time with tools*. (B-5-3)

In summary, the knowledge required for hand stitchers is the domain-specific procedural knowledge to understand the different processes of hand stitching. In terms of skills, hand stitchers, in addition to having natural talent and dexterous hands, must also have the manual skills to use needles and threads for hand stitching, i.e., adopt and manage objects, a set of tools or devices, and artefacts to achieve specific results (Organization for Economic Co-operation and Development, 2019b). In addition, the skills of carefulness and perseverance enable hand stitchers to work long hours and have perseverance, so that individuals will try their best to accomplish their preserving goals even if they encounter setbacks (Taguma and Anger, 2016).

### Bag production supervisors’ professional literacies

**Figure 6:**
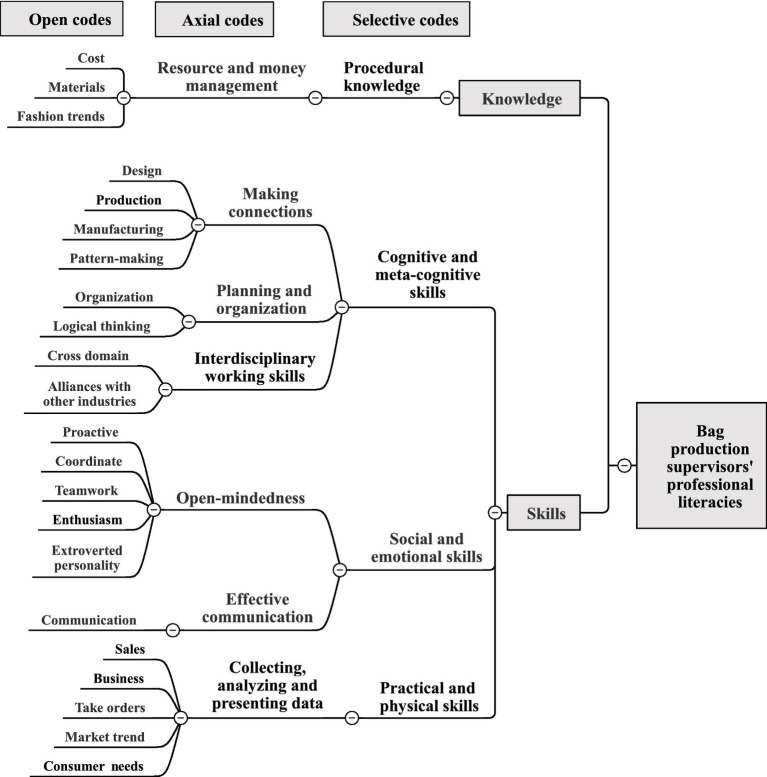
Bag production supervisors’ professional literacies coding.

The following is an analysis of professional literacies that should be possessed by production supervisors in Taiwan’s bag manufacturing industry (see Figure 6).

In terms of knowledge, since Taiwan’s bag manufacturing industry has to control materials and costs very strictly, production supervisors should have procedural knowledge of resource and money management (e.g., materials, costs, and fashion trends). Interview comments include:

*They should require familiarity with material characteristics, and knowledge of bag trends.* (A-6-1)

*Production supervisors must understand fashion trends and control materials and costs very strictly*. (C-6-10)

*To know material characteristics and fashion trends and cost control management and at what price you want the goods to be sold*. (G-6-3)

In terms of skills, production supervisors are required to have the following three skills:

1. Cognitive and meta-cognitive skills: First of all, in order to facilitate the establishment of good contacts with various functions, if they have bag design, pattern-making, and manufacturing skills, they have the ability to add wings to the management, monitoring, and coordinating of the daily production activities of the manufacturing industry, supervising quality control and managing the bag production staff. Secondly, they should have interdisciplinary working skills for alliances with other industries; finally, they should have logical thinking and organizational skills as the job also involves organizing workflow and handling production schedules and costs. Interview comments include:

*Production supervisors who have skills in bag design, pattern-making and manufacturing will be able to find problems on the job at any time, or when employees have problems, they can also help solve them immediately*. (A-6-4)

*Production supervisors have strong logical thinking and organizational skills, and many people are born with this potential, so they can use it in a timely manner*. (A-6-6)

*Production supervisors must know everything, because the bag manufacturing industry crosses a lot of industries; they also need to have interdisciplinary working skills for cross-industry alliance*. (D-6-4)

2. Social and emotional skills: production supervisors should have open-mindedness (e.g., an outgoing personality, positive, enthusiastic, coordinated, and team player) to communicate well with colleagues and customers. Interview comments include:

*A relatively outgoing person who describes the benefits of their products to the outside world and enjoys marketing or media exposure, who can be a competent production supervisor*. (E-6-11)

*Production supervisors should have a positive and enthusiastic attitude toward dealing with people*. (A-6-7)

*Of course we will work as a team, we will discuss and coordinate with each other and share our experience*. (E-6-11)

3. Practical and physical skills: production supervisors must have the ability to collect, analyze, and present data (e.g., business, sales, order taking, consumer demand, cross-industry integration, composite material integration, and market trends). Interview comments include:

*In addition to the professional technology of pattern-making, design and sewing, we should also have the ability to develop and design, and understand the changes and pulse of the bag market in order to know the needs of customers and design bags that meet the needs of the market*. (A-6-1, A-6-2)

*It is important to understand the changing market trends of bags, cross-industry combination, and composite material combination*. (A-6-3)

*Make bags that meet the needs of consumers, not make ones that you think are good*. (E-6-11)

*The most important thing is sales, how the business receives orders*. (E-6-11)

To sum up the above, the knowledge required by production supervisors is procedural knowledge of resource and money management, which is also classified as financial literacy, which helps to reduce production costs (Xu and Zia, 2012). In terms of skills, because production supervisors need to link with other job workers, they must have a variety of professional knowledge and the ability to enrich themselves at any time by learning-to-learn, for meta-cognitive skills. Production supervisors in the manufacturing industry’s daily production activities face complex problems when finding solutions (Organization for Economic Co-operation and Development, 2019b). The logical thinking skills are the cognitive skills of higher order thinking skills, and there is still insufficient evidence to prove whether having this skill can improve productivity or achievement (Taguma and Anger, 2016). Social and emotional skills related to open-mindedness, such as collaboration and communication, enable production supervisors to better define personal targets, handle personal emotions, strengthen interpersonal connections and ensure productivity (Payton et al., 2008).

In addition, with influential communication, all participants can reach their targets, at least partially or during the interaction (Taguma and Anger, 2016), and people with outgoing and enthusiastic personalities are insightful, respectful, and considerate of others (Berger and Frey, 2015). In addition, the ability to collect and analyze, present skills of market requirement, consumer demand, business, sales, order taking, market research, interviews or big data analysis are necessary for digital literacy. This is the key competence for lifelong learning, which includes information and data literacy, collaboration and communication, digital formation innovation, security, and problem solving (Vuorikari et al., 2016).

## Conclusion and recommendations

### Conclusion

This study defines professional literacy as professional competence, covering knowledge, skills, and attitudes and values. According to the purpose of this study, Taiwan’s bag manufacturing industry talents were explored. In the bag manufacturing area or profession, extensive professional knowledge, skills, and attitudes and values are required. The conclusions of this study are described below according to the results of the study.

From a knowledge perspective, the common professional literacies that should be possessed by Taiwan’s bag manufacturing industry talents are procedural knowledge, which are also required by bag designers, manufacturing technicians, patternmakers, hand stitchers, and production supervisors. This is because procedural knowledge is the domain-specific knowledge of the bag industry, which is used in contrasting subjects and conditions to distinguish results and overcome problems (Organization for Economic Co-operation and Development, 2019a). With this procedural knowledge framework, one can develop systems thinking and design thinking and then describe as an action of strategy, production and internalization, to solve complex problems that are difficult to define (Mobus, 2018; Organization for Economic Co-operation and Development, 2019a).

From the skills perspective, the common professional literacies that should be possessed by Taiwan’s bag manufacturing industry talents are cognitive and meta-cognitive skills, and social and emotional skills. This is also a professional literacy that bag designers, patternmakers, and production supervisors should possess; manufacturing technicians must also have social and emotional skills. On the other hand, bag designers, manufacturing technicians, patternmakers, hand stitchers, and production supervisors should have both practical and physical skills, as described below:

1. Cognitive and meta-cognitive skills: In addition to emphasizing the need for patternmakers to have self-regulation and learning-to-learn skills for autonomous learning, this study also advocates that Taiwan’s bag manufacturing industry talents should have the ability to integrate cross-disciplinary expertise, competencies, and resources to lead the operations of a company or organization. This result supports the point made by Burciul and Sloan (2013) that cultivates and reinforces them to turn into transformative leaders, navigating transformation and building an energetic social influence in their communities. In addition, Gudanowska et al. (2020) pointed out that adapting, designing, framing, futuring, scanning, and visioning are constructed as a foresight maturity model and also as a foresight competency model. That is to say, bag designers, patternmakers, and production supervisors may not be in charge of Taiwan’s bag manufacturing industry, or the actual leaders, but they must have a vision, a disruptive future, and engage others in their responsibilities by reaching worthwhile targets and applying predominant strategies over time (Taguma and Anger, 2016).
2. Social and emotional skills: The findings of this study highlight the importance of communication and collaboration. Nowadays, social emotional learning is becoming increasingly important in both education and the workplace, empowering people to beneficially determine their targets, handle their emotions, strengthen their interpersonal connections, and improve their achievement expression, emotional acceptability, emotional regulation, sufficient communication, decision-making, goal-setting, empathy and problem resolving (Payton et al., 2008). In addition, the competence of collaboration and working in a team help to absorb knowledge/information from others, understand others’ demands, opinions and conduct, manage team conflict, facilitate collaboration and participatory problem solving, and through collaboration, one can build one’s courage or motivation and ultimately have an impact worldwide and make a significant impact through a part of it (Taguma and Anger, 2016). In addition, people with outgoing, enthusiastic, and insightful personalities are respectful and considerate of others (Berger and Frey, 2015), have boundless enthusiasm for their work, are innovative, are willing to experiment, and have the courage to learn from their failures (Galvin, 2015).
3. Practical and physical skills: Since the purpose of this study was to probe professional literacy that Taiwan’s bag manufacturing industry talents should possess, practical and physical skills are very important in the minds of bag manufacturing industry leaders or supervisors. Bag designers, manufacturing technicians, patternmakers, hand stitchers, and production supervisors are required to possess the practical and physical skills required for the occupation. For example, bag designers, manufacturing technicians, patternmakers and hand stitchers should possess manual skills, which help with the use of objects, a set of tools or devices, and artefacts to achieve specific results (Organization for Economic Co-operation and Development, 2019b). On the other hand, bag designers, manufacturing technicians and patternmakers should also have the skills to use the newest ICT devices in order to cope with the era of automation and information. In addition, due to the different job attributes, manufacturing technicians should have persistence and repeated practice skills to enhance their professional skills, and also make them understand that it is necessary to make an effort to persist after failure, and be able to produce success (Pourdehnad et al., 2011). Hand stitchers need to have the skills of care and perseverance so that they can work long hours and have the perseverance so that they will try to accomplish their goals even if they encounter setbacks (Taguma and Anger, 2016). Production supervisors should have digital literacy to facilitate market research, interviews, or big data analysis.

In terms of attitudes and values, social values are related to interpersonal relationships and working efficiently, which are essential for developing knowledge and skills (Organization for Economic Co-operation and Development, 2019c). Social skills are also important skills for promoting social capital and social well-being (Schoon and Haste, 2018). Taiwan’s bag manufacturing industry talents are considered to have social values. Their social values are innovative thinking, change and diversification, flexibility, adaptability, all of which are required to adjust to high-speed technological progress. Workers must obtain social skills, as well as knowledge, attitudes and values, in order to maintain competitiveness. Workers must obtain the newest knowledge and skills throughout their working careers, which requires openness and a good attitude for long-established learning and curiosity (Berger and Frey, 2015; Schoon and Haste, 2018).

### Recommendations

This research provides recommendations for Taiwan’s bag manufacturing industry and for Taiwan’s technical and vocational education (TVE) based on the conclusion of the professional literacy that Taiwan’s bag manufacturing industry talents should possess.

#### Encourage Taiwan’s bag manufacturing industry’s employers to support related practitioners to develop professional literacy with knowledge, skills, and attitudes and values

“Performance management” provides an organization with clear objectives to provide all people with a clear direction to work towards, so that they can work together to promote and accomplish the organization’s mission. One of the important tasks of a manager in an organization is to “nurture and inspire” his or her subordinates, and the supervisor must take responsibility for assisting in the development of the subordinates’ professional literacy to achieve “performance improvement” in performance management (Amici and Cepiku, 2020). The supervisor must play a key role in identifying the knowledge, skills, and attitudes and values required for the role in the job analysis of the subordinate’s duties, and the level of competency required for each item.

In addition, social and emotional skills should also be emphasized. The Organization for Economic Co-operation and Development (2019c) suggests that AI is unlikely to substitute workers who need creativity or sophisticated social networks so as to adjust to technological progress; workers need to obtain social skills, including persuading and negotiating with others (Berger and Frey, 2015). In the face of a work environment that is increasingly dependent on complex machines, growing reliance on complex machines leads to the danger of some devaluing others, and this devaluation is already occurring (Turkle, 2017). Therefore, learning how to recognize the human value of oneself and others will become increasingly important (Putnam, 2000). Valuing people’s contributions to society is necessary not only for personal and societal well-being, but also for the health and relationship of the organization (Berkowitz et al., 2018). Therefore, it is suggested that Taiwan’s bag manufacturing industry employees can refer to the conclusion of this study to support the development of professional literacy with knowledge, skills, and attitudes and values. The government authorities can provide support based on the funding formulas that fully motivate employees to invest in learning, or employers can cooperate with other manufacturers to provide guidance on learning opportunities for relevant employees, thereby reducing costs.

#### Planning bag-related courses of technical and vocational education with the professional literacy orientation of knowledge, skills, and attitudes and values

Although this study focuses on the professional literacy that Taiwan’s bag manufacturing industry talents should possess, we cannot avoid thinking about the cultivation of bag professional and technical talents in Taiwan’s TVE. The hands-on course of the home economics group of senior high school (such as Practice of Multi-Material Creation, Practice and Design of Accessories) teaches basic bag design and production, which is the articulation to the university bag-related courses. This is a very important foundation for university bag-related education. Most of the higher education bag talents cultivation, mainly the department of fashion design, emphasizes practical experience and creative practice of bag design and production education, and curriculum planning emphasizes bag knowledge connotation and skill training cultivation.

However, in developing social and emotional skills, attitudes and values, self-discipline, communication, and problem solving must be learned through student teamwork in thematic courses (Fan and Ye, 2022). From this point of view, university departments offering bag-related courses should avoid the “bag-learning for dummies” mentality of quick learning in their curriculum planning and teaching, and should strengthen the teaching of professional knowledge, as well as cultivating social and emotional skills, and attitudes and values, to enhance the overall professional literacy of bag design and production technology talents. The development of any professional knowledge takes a long time to accumulate, while social and emotional skills, and attitudes and values need to be deeply experienced through practice.

In summary, it is suggested that the bag design and production education of TVE in Taiwan should practice competency-based education, which not only echoes the conclusion of this study, but also supports the viewpoints of related studies. For example, Le Deist and Winterton (2005) suggested that competency-based education should include knowledge concepts, experiences, and behaviors, as opposed to the action competence of traditional German technological and vocational education. Moreover, emphasizing subject area knowledge, cross-contextual skills and methods, it is argued that personal, social, cognitive, and methodological competence should be integrated across educational stages (Brockmann et al., 2008). Gulikers et al. (2009) argued that the characteristics of competence should include: (1) the integration of a cluster of knowledge, skills and attitudes; (2) the demonstration of effective problem solving in specific areas of expertise, organizations, jobs, roles, situations, and tasks; (3) embedded in specific contexts and given meaning and level of achievement by specific work contexts; and (4) demonstrated through behavioral or task orientation.

## Limitations and further study

This study established that professional literacy that Taiwan’s bag manufacturing industry talents should possess is applicable for Taiwan’s bag manufacturing industry designers, manufacturing technicians, patternmakers, hand stitchers, and production supervisors. However, this study did not ask about the professional literacy required for the relevant duties of professionals in the bag manufacturing industry’s raw material production and sales, suggesting that future research might interview professionals in raw material production and sales to enhance the professional literacy that Taiwan’s bag manufacturing industry talents should possess. Secondly, this study mainly focused on Taiwan’s bag manufacturing industry executives or senior managers, but there are still limitations of other minority groups. Thus, this is the second limitation of this study; therefore, in the future, the work of others in the bag manufacturing industry can be studied and investigated separately, such as designers, manufacturing technicians, patternmakers, hand stitchers, and production supervisors, or other workers, to avoid biased data.

In addition, subsequent researchers can also use the modified Delphi method proposed by Murry et al. (1995) to conduct a large scale expert Delphi method survey by combining structured questions with the traditional Delphi method of open-ended questionnaires. This would allow participation and weighting of several experts. The results of this study can be extended vertically and horizontally to make the theory more saturated and solid and to enhance the depth and breadth of the study, making it more academically valuable and offering practical contributions.

## Data availability statement

The original contributions presented in the study are included in the article/supplementary material, further inquiries can be directed to the corresponding author.

## Ethics statement

Ethical review and approval was not required for the study on human participants in accordance with the local legislation and institutional requirements. The interviewees provided their written informed consent to participate in this study.

## Author contributions

J-YF confirms being the sole contributor of this work, who contributed to collect and prepare the data, analyzed the data, conceptualized and wrote and revised the draft, and has approved the submitted version of the draft.

## Conflict of interest

The author declares that the research was conducted in the absence of any commercial or financial relationships that could be construed as a potential conflict of interest.

## Publisher’s note

All claims expressed in this article are solely those of the authors and do not necessarily represent those of their affiliated organizations, or those of the publisher, the editors and the reviewers. Any product that may be evaluated in this article, or claim that may be made by its manufacturer, is not guaranteed or endorsed by the publisher.
